# Calorie Restriction Mimetics: Upstream-Type Compounds for Modulating Glucose Metabolism

**DOI:** 10.3390/nu10121821

**Published:** 2018-11-22

**Authors:** Hideya Shintani, Tomoya Shintani, Hisashi Ashida, Masashi Sato

**Affiliations:** 1 Department of Internal Medicine, Saiseikai Izuo Hospital, Osaka 551-0032, Japan; sntnhdy@gmail.com; 2 United Graduate School of Agricultural Science, Ehime University, Matsuyama 790-8577, Japan; 3 Faculty of Biology-Oriented Science and Technology, Kindai University, Wakayama 649-6493, Japan; ashida@waka.kindai.ac.jp; 4 Faculty of Agriculture, Kagawa University, Kagawa 761-0701, Japan; sato@ag.kagawa-u.ac.jp

**Keywords:** calorie restriction mimetics, antiaging, lifespan extension, glucose metabolism modulation, chitosan, acarbose, SGLT2 inhibitor, 2-deoxy-d-glucose, d-allulose, d-glucosamine

## Abstract

Calorie restriction (CR) can prolong the human lifespan, but enforcing long-term CR is difficult. Therefore, a compound that reproduces the effect of CR without CR is needed. In this review, we summarize the current knowledge on compounds with CR mimetic (CRM) effects. More than 10 compounds have been listed as CRMs, some of which are conventionally categorized as upstream-type CRMs showing glycolytic inhibition, while the others are categorized as downstream-type CRMs that regulate or genetically modulate intracellular signaling proteins. Among these, we focus on upstream-type CRMs and propose their classification as compounds with energy metabolism inhibition effects, particularly glucose metabolism modulation effects. The upstream-type CRMs reviewed include chitosan, acarbose, sodium-glucose cotransporter 2 inhibitors, and hexose analogs such as 2-deoxy-d-glucose, d-glucosamine, and d-allulose, which show antiaging and longevity effects. Finally, we discuss the molecular definition of upstream-type CRMs.

## 1. Introduction

Some records indicate that the first emperor of Qin ordered a search for an elixir of immortality. There is also a legend stating that Egyptian pharaohs most desired an antiaging immortality medicine. Even in Japan, one of the world leaders in longevity [1], new information on health and longevity is easily accessible from the media, as many people seek for knowledge on this subject. Indeed, people worldwide have been searching for longevity and immortality since ancient times.

In the 2000s, calorie restriction (CR) was shown to extend the lifespan of rhesus macaques, which are primates in the same order as humans [2]. However, a subsequent report [3] indicated that CR promoted health but did not extend lifespan. Because these studies did not use the same dietary conditions, it was difficult to directly compare the results [4]. Additionally, in primates, CR was suggested to extend lifespan depending on dietary conditions. Although a series of reports represented landmark achievements, it is difficult for people to implement CR in the same manner. In recent years, studies have progressed to searching for and evaluating compounds showing effects similar to those of CR upon oral administration.

## 2. Calorie Restriction

**Figure 1:**
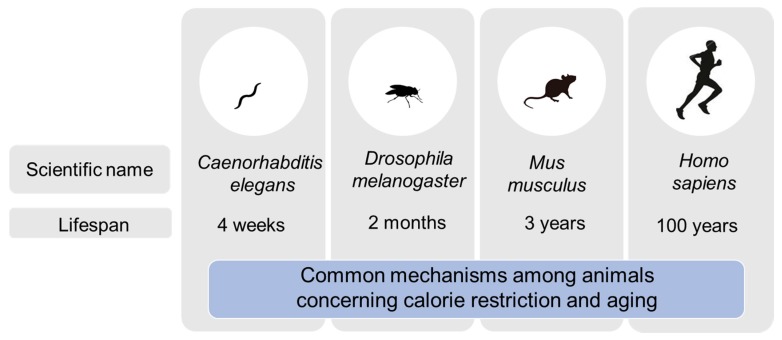
Experimental animal models.

CR has been shown to prolong the lifespan of experimental animal models such as nematodes, flies, and mice (Figure 1). CR delays the progression of diverse age-related changes and diseases. The Comprehensive Assessment of Long-Term Effects of Reducing Intake of Energy (CALERIE) was the first study that focused on the effects of CR in humans [5,6]. The CALERIE demonstrated the feasibility of CR (for at least two years) in humans and its favorable effects on predictors of longevity and cardiac metabolic risk factors [7]. Previous clinical studies of nonobese subjects did not achieve the extent of CR or sustained weight loss observed in the CALERIE trial. Other notable features of the CALERIE were the size of the clinical trial; comprehensive physiological examinations; psychological, quality of life, and cognitive assessments; and extensive biochemical tests of the serum, plasma, and urine.

The CALERIE trial was conducted in two stages. Phase 1 was a preliminary study for determining the target percentage of CR, while Phase 2 tested the effects of CR in a two-year randomized clinical trial. A longer intervention period resulted in an extended CR effect that persisted after the acute effect on weight loss. The specific primary purpose of the CALERIE Phase 2 was to test the hypothesis that human CR causes persistent metabolic adaptation. The Phase 2 trial, completed in 2013, was a two-year, three-site randomized controlled trial in young and middle-aged nonobese healthy males and females. The results for the group assigned to different levels of CR were compared to those in the optional meal management group. Among the 53 nonobese subjects, 34 were included in the calorie-restricted group, with 15% of calorie intake restricted for two years, while the remaining 19 had free access to food. As a result, in the CR group, weight loss, body fat reduction, fluctuations in energy consumption, and decreased oxidative stress markers were observed [7]. These changes were also observed in experimental animal models of CR.

## 3. Calorie Restriction Mimetics

Even if CR prolongs the human lifespan, it is difficult to enforce long-term CR in humans. Therefore, it is preferable to develop a method or compound that reproduces the effect of CR without limiting the amount of food. The concept of CR mimetics (CRMs) was proposed by Lane et al. [8] in a study of 2-deoxy-d-glucose (2DG), which showed bioactivity in rats. CRMs exhibit the systemic effects of CR and broadly include not only compounds but also methods such as bariatric surgery or exercise [8,9].

Downstream and upstream CRMs have been identified [10,11,12,13]. Downstream-type CRMs act on an intracellular signaling system and exert the same effect as CR on downstream pathways (Table 1). In contrast, an upstream-type CRM uses a mechanism of action targeting the energy metabolism system and transmitting a signal in the upstream direction to mimic CR (Table 2). In fact, it is difficult to strictly classify all CRMs as belonging to either type, and therefore, a molecular definition of CRMs is required [14]. From the current perspective, we selected 11 CRM candidates for which more evidence is available and discuss their characteristics, including a comparison of downstream- and upstream-type CRMs. Here, we propose that upstream-type CRMs should be classified as compounds with energy metabolism inhibition effects, particularly glucose metabolism modulation effects.

### 3.1. Downstream-Type Calorie Restriction Mimetics

#### 3.1.1. Metformin

Metformin is a representative drug for treating diabetes and is recommended as the first-line drug for type 2 diabetes in the guidelines of the American Diabetes Association and European Association for the Study of Diabetes [15,16]. At least some of the effects of metformin are mediated by AMP-activated protein kinase (AMPK) [17]. Metformin transiently inhibits the mitochondrial respiratory chain (specifically, complex I), increases the intracellular AMP/ATP ratio, and activates AMPK. AMPK generally promotes catabolic reactions that produce ATP and suppresses anabolic reactions that consume ATP. In the liver, gluconeogenesis and fatty acid synthesis are suppressed, while β-oxidation is promoted. In the skeletal muscles and adipose tissue, AMPK promotes the translocation of glucose transporter type 4 to the cell membrane and stimulates sugar uptake [18]. Although the mechanism is unknown, suppression of sugar absorption from the intestinal tract may contribute to the hypoglycemic effects of metformin [19].

**Figure 2:**
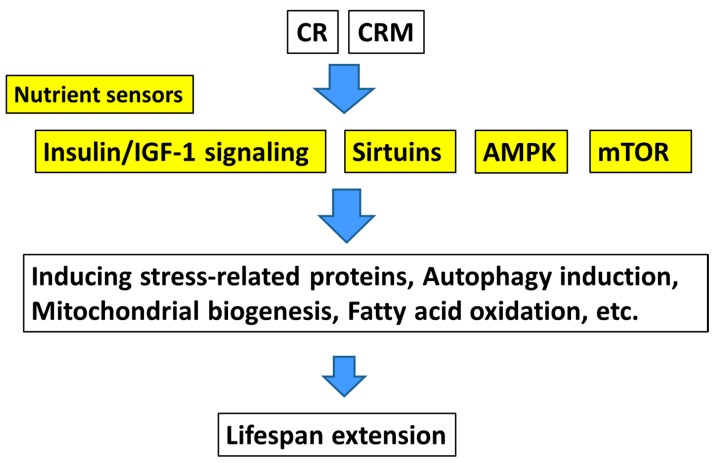
Postulated mechanisms of action of CR and CRMs.

Administration of metformin to experimental animals results in different effects depending on the animal species. In the nematode *Caenorhabditis elegans*, metformin slowed the accumulation of lipofuscin, extended the median lifespan, and prolonged a youthful locomotory ability in a dose-dependent manner [20]. The mechanism of prolonging the lifespan of *C. elegans* by metformin may include alterations in microbial folic acid and methionine metabolism [21]. It was also shown in *C. elegans* that metformin may prolong the lifespan through a mitochondrial process and that the production of metformin-induced reactive oxygen species (ROS) induced a signaling cascade that increased the overall lifespan [22]. In *C. elegans*, both target of rapamycin complex 1 (TORC1) inhibition and AMPK activation contributed to the lifespan-extending effect of metformin [23] (Figure 2). Genetic screening of *C. elegans* revealed that metformin was inactivated by inactivation of TORC1, a mechanism conserved from invertebrates to humans [22]. In *Drosophila*, administration of metformin caused activation of AMPK and reduced the weight of adipose tissue; however, the drug did not prolong lifespan [24]. Long-term intake of metformin with a starch-supplemented diet shortened the lifespan of *Drosophila melanogaster* compared to the group fed the starch-supplemented diet alone [25]. In C57BL/6 mice administered two doses of metformin (0.1% and 1%), the survival rate was significantly reduced at a higher dose compared to in the control; however, at a lower dose, the survival rate significantly increased, even when the mice consumed more feed [26].

The results of a UK prospective clinical study of metformin as a treatment for diabetes indicated a reduced total mortality, which occurred independently of glycemic control [27]. A systematic review and meta-analysis of 260 articles by Campbell et al. [28] indicated that diabetics administered metformin had a significantly lower all-cause mortality compared to nondiabetics and diabetics administered non-metformin therapies. Additionally, metformin users had a lower incidence of cancer than did nondiabetic subjects, and the incidence of cardiovascular disease was reduced compared to that in patients with diabetes administered non-metformin therapy. The clear decrease in aging associated with metformin use suggests that metformin prolongs life and improves health by acting as an antioxidant.

#### 3.1.2. Rapamycin

Rapamycin (International Nonproprietary Name: sirolimus) is a macrolide compound produced by *Streptomyces hygroscopicus* that was first isolated on Easter Island in 1972 [29]. This compound is the most widely used inhibitor of mammalian target of rapamycin (mTOR). Rapamycin exerts substantial regulatory effects on important biological processes such as proliferation and inflammation [30]. Particularly, because of its ability to inhibit immune responses, rapamycin has been used clinically to prevent transplant rejection and treat autoimmune diseases [31]. Rapamycin inhibits interleukin-2 signaling and other cytokine-receptor-dependent signaling pathways by acting on mTOR and prevents the activation of T and B cells. Its mechanism of action, which is similar to that of tacrolimus, involves binding to the cytoplasmic protein FK-binding protein 12 (FKBP12). However, unlike the tacrolimus–FKBP12 complex which inhibits calcineurin (also known as protein phosphatase 2B), the rapamycin–FKBP12 complex inhibits the mTOR pathway by binding directly to mTOR complex 1 [32].

Robida-Stubbs et al. [33] showed that SKN-1/nuclear respiratory factor and DAF-16/forkhead box protein O activated protective genes and increased stress tolerance and lifespan when TORC1 was genetically inhibited in *C. elegans*. In this study, the authors showed that the mean lifespan of nematodes treated with rapamycin increased by 19%. Another recent study revealed that decreased expression of the CCAAT-enhancer-binding protein β (C/EBPβ) LIP isoform, stimulated by mTOR complex 1, delayed the development of age-related phenotypes in mice, making C/EBPβ-LIP a new antiaging target [34]. The mTOR pathway, along with sirtuin family proteins and the insulin/insulin-like growth factor signaling pathway, is an important pathway that regulates lifespan [35] (Figure 2). In a *Drosophila sod1* (superoxide dismutase 1 gene) mutant, rapamycin extended the average lifespan by 6% in males and 26% in females maintained on standard feed [36], although the data were inconsistent when low-calorie feed or high-sugar/low-protein feed was provided. Similarly, rapamycin promoted survival in a *Drosophila* model of a mitochondrial disease [37].

Rapamycin prolonged the lifespan of mature mice by 28–38% compared to control animals [38]. Because these mature mice were 20-months old, corresponding to approximately 60 years of age in humans, the data suggest that life expectancy can be extended in already aged humans. In another report of middle-aged mice, three months of rapamycin treatment increased the average lifespan by 60%, improving the health status of middle-aged and elderly mice [39]. Low-dose rapamycin also extended the lifespan of mouse models with mitochondrial disorders [40]. Additionally, it was shown that rapamycin treatment had a beneficial effect on the arterial function of old mice, and these improvements were associated with decreased expression of proteins involved in oxidative stress, AMPK activation, and cell cycle control [41]. Another study showed that rapamycin had beneficial effects on neoplastic diseases, delayed multiple aspects of mouse aging, and extended longevity [42]. However, the incidences of testicular degeneration and cataract were significantly higher in rapamycin-treated mice, depending on the dose and timing of rapamycin administration. As side effects of long-term administration of rapamycin, hyperglycemia, impaired glucose tolerance, and insulin resistance have also been reported in studies using rats [43].

#### 3.1.3. Resveratrol

Resveratrol is a natural polyphenolic phytoalexin mainly present in the skin of grapes and in red wine [44]. This polyphenol has been most thoroughly studied as a compound that activates sirtuin 1 or its invertebrate homologs [45]. Resveratrol is known to protect living organisms against ROS and was shown to exert its antioxidant effects by activating SIRT2 to deacetylate peroxiredoxin 1 [46]. These data theoretically explain how red wine reduces the health risks associated with unhealthy meals, thus explaining the French paradox. Resveratrol has been reported to prolong the lifespan of several different species; however, conflicting views are increasingly expressed [47,48,49,50,51,52,53].

It has been reported that treating *C. elegans* with resveratrol prolonged its lifespan via a mechanism completely dependent on SIR-2.1 [47] (Figure 2). Resveratrol prolonged the lifespan of *C. elegans* in vivo under oxidative stress but not under normal conditions [48]. At a concentration of 400 μM, resveratrol extended the lifespan of female *Drosophila* fed a high-fat diet by 10%–15% [49]; however, the results differed depending on the feed content. In a recent study, supplementation with 500 μM of resveratrol did not affect the stress response and expression of genes associated with longevity in flies nor did it prolong the lifespan [50]. A study in which the mosquito *Anopheles stephensi* was administered resveratrol orally at various concentrations (0, 50, 100, or 200 μM) led to the conclusion that, under normal conditions, resveratrol did not prolong the life of *A. stephensi* [51]. Extension effects on the mean lifespan were observed when resveratrol was administered to obese mice induced by a high-fat diet [52]. Resveratrol also preserved indices of vascular function in normal rats, although without extending the lifespan [53].

In clinical studies, intake of resveratrol improved the memory capacity of elderly subjects and improved the blood lipid levels and glucose control in obese and adult diabetic subjects [54,55,56]. However, in one study, resveratrol improved vascular function, particularly in elderly people, but did not improve glucose metabolism [57].

#### 3.1.4. Polyamines

Longevity in mice has been shown to be promoted by gut bacterial polyamine production [58]. Spermidine, a well-studied polyamine, is present in many fermented foods such as yogurt and miso. Treatment with spermidine increased the lifespan of yeasts, worms, and flies, and studies showed that enhancement of autophagy was involved in reducing oxidative stress in these models and mice [59]. Administration of spermidine to mice prolonged the median lifespan, even in middle-aged mice [60].

Spermidine has been shown to not only exert significant cardioprotective and neuroprotective effects in rodent models but also stimulate anticancer immune surveillance. Additionally, it has been reported that polyamine consumption in food was correlated with reduced cardiovascular- and cancer-related mortality in human epidemiological studies [61].

#### 3.1.5. Other Downstream-Type Calorie Restriction Mimetics

Oxaloacetic acid is an intermediate of the Kreb’s cycle and is related to Nicotinamide adenine dinucleotide (NAD^+^) levels and redox balance in cells. In *C. elegans*, oxaloacetic acid was shown to prolong the lifespan independently of sirtuin but dependently on AMPK [62]. In a pilot clinical study, a tendency for decreased blood glucose levels was observed following administration of oxaloacetic acid [63].

### 3.2. Upstream-Type Calorie Restriction Mimetics

#### 3.2.1. Chitosan

**Figure 3:**
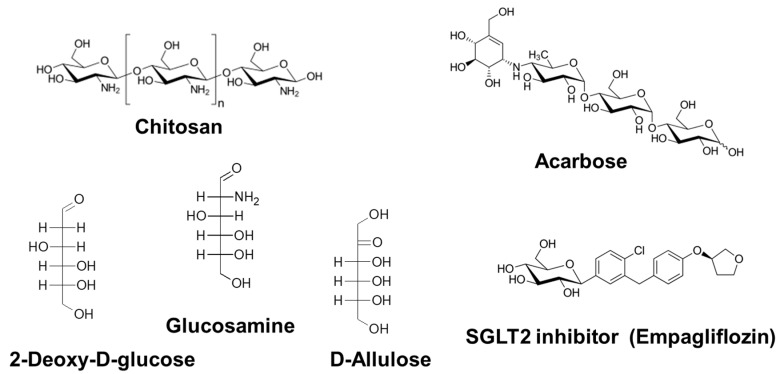
Chemical structures of upstream-type CRMs.

Chitosan is a polysaccharide composed of β1,4-linked d-glucosamine (GlcN) and *N*-acetyl-GlcN (Figure 3). It is purified by boiling chitin, obtained from the exoskeleton of crustaceans such as crab and shrimp, in concentrated alkali. The effect of chitosan intake manifests as decreases in the body weight and blood lipids and may prolong lifespan.

Examination of the effects of long-term feeding of chitosan on plasma glucose and lipids in rats fed a high-fructose diet showed that chitosan reduced plasma glucose levels and improved impaired glucose tolerance and insulin tolerance [64]. Chitosan also improved the lipid profile, insulin sensitivity, and oxidative stress in rats, which were exacerbated by a high-fat/high-cholesterol diet [65]. Chitosan has been reported to have glucose- and fat-lowering effects. Decreased intestinal disaccharidases and prolonged glucose absorption in the small intestines were suggested to be partly involved in reducing plasma glucose levels in diabetic rats [66]. Chitosan not only suppresses the absorption of lipids but may also affect the absorption of carbohydrates. Additionally, because lipid metabolism is closely related to glucose metabolism [67], the effect of chitosan as a CRM may be related to improved glucose metabolism through improved lipid metabolism.

Analysis of the data obtained from six studies, including chitosan treatment in patients with hypercholesterolemia, revealed a significant decrease in the total cholesterol level [68]. In contrast, chitosan had no significant effect on lipid fractions. However, several conflicting reports were subsequently published. Chitosan administered for four months to 28 patients with plasma triglyceride levels of >150 mg/dL reduced total cholesterol by 8%, low-density lipoprotein (LDL) cholesterol by 2%, and triglycerides by 19% and increased high-density lipoprotein cholesterol by 14% [69]. In a clinical trial in which 3200 mg of chitosan was administered to 116 obese patients for 12 weeks, serum LDL cholesterol significantly decreased in the chitosan treatment group [70]. Recently, a systematic review of the effects of chitosan reported reduced total cholesterol, very-low-density lipoprotein cholesterol, and LDL cholesterol [71]. Additionally, a meta-analysis of eight trials involving 600 participants showed that short-term (<12 weeks) chitosan intake at a high dose (>2.4 g/day) significantly reduced diastolic blood pressure [72].

#### 3.2.2. Acarbose

Acarbose, an α-glycosidase inhibitor, significantly increased the median and maximum lifespans of male mice at a 1% concentration [73]. In rats, acarbose reduced body weight and body fat and improved glucose metabolism without reducing food intake [74]. Similar to metformin, acarbose is used to treat diabetes worldwide and is thus guaranteed to be highly safe and efficacious. Therefore, its effects on longevity can be analyzed in healthy subjects. Acarbose is well known to suppress blood glucose levels in humans [75,76]. A post hoc analysis found that acarbose treatment reduced body weight independently of the glycemic control status but dependently on the baseline body weight [77].

#### 3.2.3. 2-Deoxy-d-Glucose

2DG is a glucose derivative in which the 2-hydroxyl group is replaced by a hydrogen atom (Figure 3). 2DG is not metabolized via glycolysis and was the first proposed dietary restriction mimetic [78]. It is thought to delay age-related dysfunctions and extend the lifespan by suppressing glycolytic activity [10]. Schulz et al. [79] proposed a detailed mechanism for the 2DG longevity effect in *C. elegans* based on a hypothetical concept named as “mitochondrial hormesis” or “mitohormesis”. The concept proposes that induction of mitochondrial metabolism may induce a positive response to increased formation of ROS and other related stressors, leading to a secondary (i.e., hermetic) increase in stress defense, resulting in reduced net stress levels. Reduction of glycolysis by 2DG induces the utilization of stored fat and mitochondrial respiration via AMPK. 2DG has been used in many studies focused on the impact of reduced metabolic rates and showed potential as a CRM. For example, in a study comparing the effects of 2DG and CR in rodents, 2DG administration showed the same effects on locomotory activity, heart rate, and blood pressure as CR administration [80].

The same group demonstrated the protective action of 2DG against glutamate excitotoxicity and upregulation of heat shock protein 70 and glutamate-responsive protein-78, which are stress response proteins, in fetal hippocampal cells [81]. Moreover, the same group demonstrated an improved behavioral outcome of 2DG treatment and reduced degeneration of dopaminergic neurons in a Parkinson’s disease model [82], as well as decreases in proliferating cell nuclear antigen and bromodeoxyuridine-positive tumor cells [83].

Although 2DG shows the same effects as CR, few studies have examined its ability to extend lifespan. Rather, long-term 2DG ingestion induced heart vacuolation in rats and increased mortality [84].

#### 3.2.4. d-Glucosamine

d-Glucosamine (GlcN; 2-amino-2-deoxy-d-glucose) is a constitutional unit of chitosan and chitin, which are produced in nature by arthropods, fungi, and cephalopods (Figure 3). GlcN is industrially manufactured by the hydrolysis of crustacean exoskeletons, which are mainly composed of chitin. GlcN is a popular dietary supplement that effectively prevents and treats osteoarthritis in humans [85].

Recently, Weimer et al. [86] reported the longevity effects of GlcN in nematodes and mice. The authors suggested that these effects were caused by impaired glucose metabolism. In contrast, Shintani et al. showed that the longevity effect of GlcN required an autophagy gene; however, the longevity genes *sir-2.1* and *daf-16* were not required for GlcN-induced lifespan extension, unlike that induced by other CRMs and CR. Similar to 2DG, GlcN enters into cells through glucose transporters and inhibits glycolysis, inducing the metabolism of stored fat and mitochondrial respiration via AMPK. Increased respiration can cause temporary formation of ROS, leading to increases in antioxidative enzyme activity, oxidative stress resistance, and survival rates [79,87]. Orally administered GlcN has also been reported to affect carbohydrate metabolism and reduce body fat in rodents [88] and contribute to enhanced oxidative stress resistance, followed by AMPK activation [86]. The compound has also been reported to induce autophagy in mammalian cells via an mTOR-independent signaling pathway [89]. Therefore, the mechanism of the antiaging effects of GlcN may be partially similar to that of 2DG.

In a clinical trial, oral administration of GlcN improved vascular endothelial function by modulating the intracellular redox state [90]. According to a large-scale epidemiological study on consumers of various dietary supplements, the use of GlcN was associated with a decrease in total mortality [91].

#### 3.2.5. d-Allulose

d-Allulose (d-Alu; d-psicose), a C-3 epimer of d-fructose, is a rare hexose sugar present in a limited quantity in nature (Figure 3). However, this compound is marketed as a functional sweetener with zero calories [92] and is easy to produce at high yields from d-fructose [93]. In the previous decade, numerous studies showed that d-Alu exhibits various activities, such as antihyperglycemic and antiobesity effects [94,95,96,97,98].

Recently, Shintani et al. [99] reported that long-term administration of a rare sugar syrup containing d-Alu maintained glucose tolerance and insulin sensitivity in rats via hepatic glucokinase translocation. Thus, d-Alu is expected to be a potent antidiabetic and antiobesity sweetener. We also reported that a high dose of d-Alu suppressed increases in body size during the young adult stage of the nematode *C. elegans* [100]. Recently, d-Alu was reported to extend the lifespan of nematodes [87]. Similar to GlcN and 2DG, d-Alu enters cells through glucose transporters and inhibits glycolysis, inducing the metabolism of stored fat and mitochondrial respiration via AMPK. Increased respiration causes temporary upregulation of ROS production, leading to increased antioxidant activity, oxidative stress resistance, and survival rates [101]. Body fat reduction was observed in *C. elegans* [87]. More recently, it was reported that d-Alu suppresses carbohydrate oxidation and promotes fat oxidation in rodents [102], supporting the above *C. elegans* model data [87].

In a clinical trial, d-Alu was shown to be a CRM based on changes in biomarker levels such as glucose and body fat. Clinical trials using a maltodextrin diet or standard meal confirmed that d-Alu suppresses postprandial blood glucose levels [103,104]. Syrup containing d-Alu showed a low glycemic response in healthy humans [105]. Even a single dose of d-Alu was reported to enhance postprandial fat oxidation in healthy humans [106]. Upon continuous intake of d-Alu, the percentage of body fat and body fat mass significantly decreased, with no significant reduction in nutrient intake [107].

#### 3.2.6. Sodium-Glucose Cotransporter 2 Inhibitors

Sodium-glucose cotransporter 2 (SGLT2) inhibitors such as empagliflozin and canagliflozin are recently approved diabetes drugs. Their mechanism of action involves inhibition of SGLT2 in the proximal renal tubules of the kidney and promotion of urinary glucose excretion by inhibiting glucose reabsorption [108]. This unique mechanism of action not only reduces plasma glucose but also has other beneficial effects, such as weight loss and blood pressure lowering [109,110,111].

In a study investigating the effects of the SGLT2 inhibitor dapagliflozin in Western-diet-induced obese mice, dapagliflozin attenuated the increase in body weight, plasma glucose, oxidative stress, and plasma triglycerides [112]. No survival time was described in the latter study; however, in another study, another SGLT2 inhibitor, bexagliflozin, prolonged the survival of rats prone to stroke [113].

In the EMPA-REG OUTCOME trial, addition of empagliflozin to standard therapy for type 2 diabetic patients at high risk of cardiovascular events reduced the incidences of cardiovascular disease deaths, cardiovascular events, and total deaths [114]. The CANVAS trial showed similar results as canagliflozin and strongly suggested that inhibition of cardiovascular events is a class effect for SGLT2 inhibitors [115].

Although SGLT2 inhibitors have not been tested in terms of prolonging the lifespan of healthy adults, they are expected to be effective as CRMs for altering biomarker levels such as body weight, blood pressure, and plasma glucose level [116].

## 4. Discussion and Summary

Since ancient times, human beings have sought longevity. Twenty years have elapsed since Lane et al. [3] proposed the CRM concept in 1998. Studies of CRMs, a long-lived “secret medicine”, have progressed greatly in the past 20 years. During this time, new candidates such as d-Alu and GlcN, both of which contain a functional hexose with high safety and health benefits, have been suggested to exert these effects. Additionally, in the current review, we described the SGLT2 inhibitor as a novel CRM candidate. Commonalities have been observed between CR and upstream-type CRMs (Table 3), which showed similar effects on selected biomarkers in experimental animal models and in humans. However, additional studies are needed to determine the mechanisms and efficacies of CRMs. CRM candidate substances that have already been studied or may arise in the future should meet certain requirements. In particular, it is necessary to confirm the life extension effect of a compound without CR and elucidate its mechanism of action as a CRM in model animals. It is also necessary to confirm that the effects of the compound on biomarkers mimic those of CR in humans.

**Figure 4:**
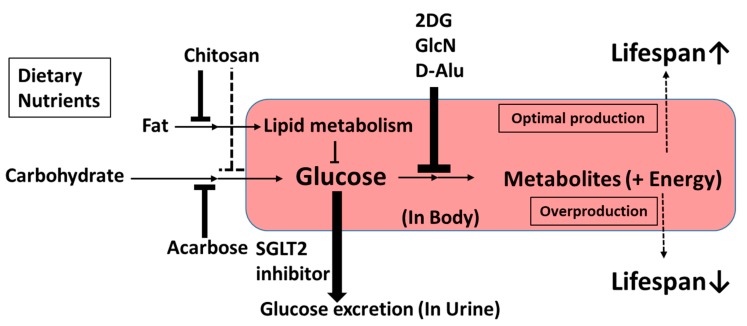
Characteristics of upstream-type CRMs.

It is also desirable to propose definitions of CRM molecules [14]. In the current review, we propose classifying upstream-type CRMs as compounds with glucose metabolism modulation effects (Figure 4). These include chitosan, acarbose, SGLT2 inhibitors, 2DG, d-Alu, and GlcN. Novel upstream-type CRMs may be found among small-molecule carbohydrate analogs, as 2DG, GlcN, and d-Alu have similar molecular structures and modulate enzymes of glycolysis, which act on d-glucose. In contrast, chitosan, acarbose, and SGLT2 inhibitors may reduce the level of d-glucose without reducing energy intake. Taken together, all CRMs of the upstream type are considered to affect glucose utilization. However, newly discovered upstream CRMs may require a novel definition in the future.

## Figures and Tables

**Table 1:** Downstream-type calorie restriction (CR) mimetics (CRMs).

Compound	Mode of Action
Metformin (antidiabetic drug)	AMPK activation
Rapamycin (immunosuppressant drug)	mTOR inhibition
Resveratrol (food component)	Sirtuin activation
Polyamines (food component)	Epigenetic control
Oxaloacetic acid (dietary supplement)	Redox balance

AMPK: AMP-activated protein kinase; mTOR: mammalian target of rapamycin.

**Table 2:** Upstream-type CRMs.

Compound	Mode of Action
Chitosan (dietary supplement)	Glucose diminution
Acarbose (antidiabetic drug)	Glycosidase inhibition
2-Deoxy-d-glucose (anticancer drug)	Glycolysis inhibition
d-Glucosamine (dietary supplement)	Glycolysis adjustment
d-Allulose (food component)	Glycolysis improvement
SGLT2 inhibitor (antidiabetic drug)	Glucose excretion

SGLT2: Sodium-glucose cotransporter 2.

**Table 3:** Commonalities between CR and upstream-type CRMs.

	Main Experimental Animal Model	Lifespan	Glucose Level	Body Weight	Body Fat	Autophagy	Energy Consumption	Stress Tolerance
CR	Human	Extend?	Lower	Lose	Decrease	Enhance in excessive CR	Change	Decrease stress markers
	Nematode/Mouse	Extend	Lower	Lose	Decrease	Enhance in excessive CR	Change	Decrease stress markers
Chitosan	Mouse	Extend?	Lower	Lose	Decrease	NR	NR	NR
Acarbose	Human/Mouse	Extend?	Lower	Lose	Decrease	NR	NR	NR
2DG	Nematode/Mouse	Extend	Lower	Lose	Decrease	Enhance	NR	Increase antioxidant enzyme
GlcN	Nematode/Mouse	Extend	Lower	Lose	Decrease	Enhance	Change	Increase antioxidant enzyme
d-Alu	Nematode/Mouse	Extend	Lower	Lose	Decrease	NR	Change	Increase antioxidant enzyme
SGLT2 inhibitors	Human/Mouse	Extend?	Lower	Lose	Decrease	NR	Change	Decrease stress markers

2DG: 2-Deoxy-d-glucose; GlcN: d-Glucosamine; d-Alu: d-Allulose; NR: Not Reported.
